# Supplementary material for: Using lidar to assess the development of structural diversity in forests undergoing passive rewilding in temperate Northern Europe
Source: PeerJ. 2019 Jan 14;6:e6219. doi: 10.7717/peerj.6219 (PMC6336013; doi:10.7717/peerj.6219)
Supplement: Supplemental Information 1 [file peerj-07-6219-s001.pdf]

PeerJ

Using lidar to assess the development of structural diversity in forests undergoing passive rewilding in temperate Northern Europe

Henrik Thers, Peder Klith Bøcher, Jens-Christian Svenning

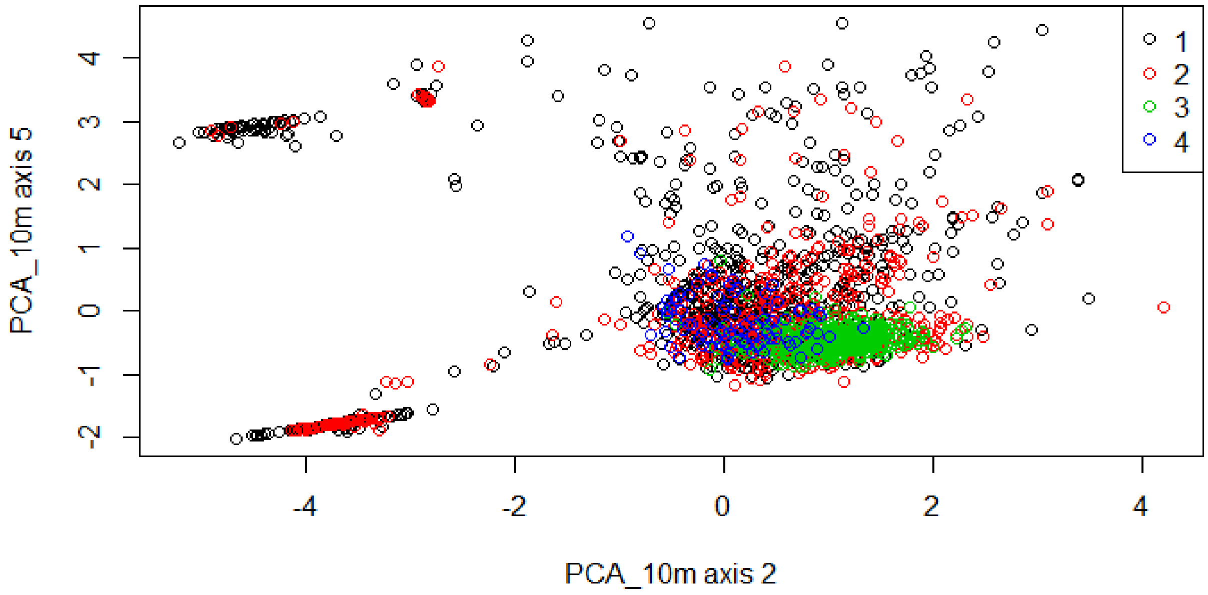

Fig. S1. Plot of the four youngest forests and zones according to axes 2 and 5 of PCA_10m.

The youngest forests and zones according to axes 2 and 5 of PCA_10m. **o**,*1: V_Y1,* o,*2: V_Y2,* o,*3: Tran_Y3,* o,*4: Fløj_Y4. Axes 2 and 5 of PCA_10m both significantly differentiate the V_Y1 and V_Y2 on both variance and medians. Despite that, the two Vorsø zones show a nearly identical distribution in the plot, which indicates that the statistical pairwise comparisons of medians and variance between areas are very sensitive to differences. This sensitivity is presumable due to different compositions of structural attributes that occur in both areas. Fløj_Y3 and Tran_Y4 are less spread reflecting a more homogenous vegetation structure. The highest values of PC2 belong to cells containing blackthorn shrub and the lowest values indicates little or no vegetation. On PC5 cells containing vegetation below one meter get the highest values and cells without vegetation the lowest values. Cells with higher vegetation get intermediate values.*
