# Supplementary material for: Using lidar to assess the development of structural diversity in forests undergoing passive rewilding in temperate Northern Europe
Source: PeerJ. 2019 Jan 14;6:e6219. doi: 10.7717/peerj.6219 (PMC6336013; doi:10.7717/peerj.6219)
Supplement: Supplemental Information 2 [file peerj-07-6219-s002.pdf]

PeerJ

# Using lidar to assess the development of structural diversity in forests undergoing passive rewilding in temperate Northern Europe

Henrik Thers, Peder Klith Bøcher, Jens-Christian Svenning

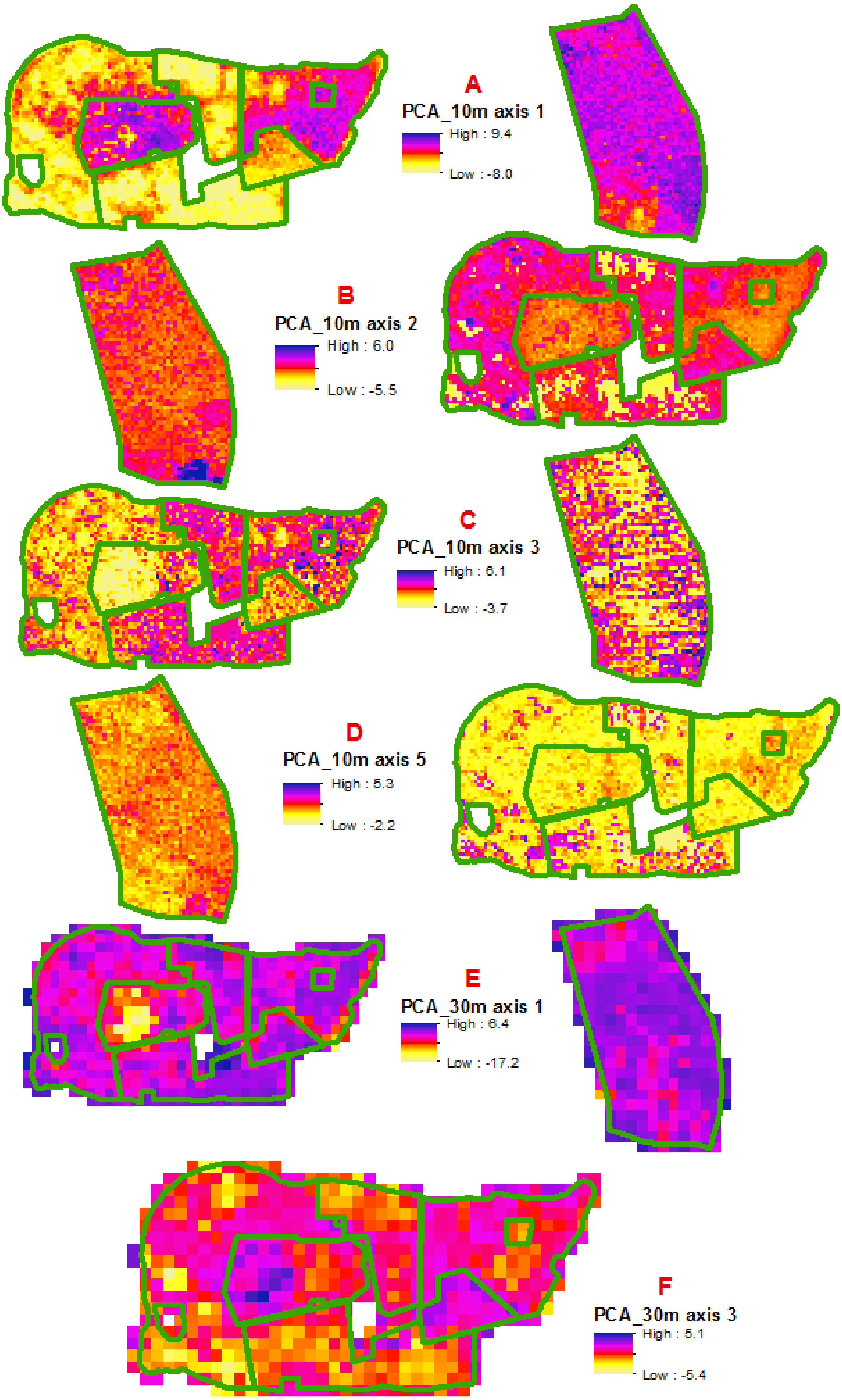
A
PCA_10m axis 1
High : 9.4
Low : -8.0
B
PCA_10m axis 2
High : 6.0
Low : -5.5
C
PCA_10m axis 3
High : 6.1
Low : -3.7
D
PCA_10m axis 5
High : 5.3
Low : -2.2
E
PCA_30m axis 1
High : 6.4
Low : -17.2
F
PCA_30m axis 3
High : 5.1
Low : -5.4

Fig. S2. Maps of selected gradients expressed by axes of PCA_10m and PCA_30m. *A: Axis 1 of PCA_10m, Vorsø to the left and Sten_M/O to the right. B: Axis 2 of PCA_10m, Sten_M/O to the left and Vorsø to the right. The highest values are for cells containing blackthorn (Vorsø) and spruce (Sten_M/O). C: Axis 3 of PCA_10m, Vorsø to the left and Sten_M/O to the right. Extremely low values are seen in the V_O1 as a result of a long-term intermediate disturbance in the old oak forest. D: Axis 5 of PCA_10m, Sten_M/O to the left and Vorsø to the right. Common patterns are seen in the V_Y1 and V_Y2 despite both medians and variance being significantly different on this axis. E: Axis 1 of PCA_30m, Vorsø to the left and Sten_M/O to the right. The unique heterogeneity of the V_O1 is indicated by the extremely low values. F: Axis 3 of PCA_30m shown for Vorsø. Cells with extreme low values indicate a unique structure composed by gaps, scrub and older trees and is limited to the V_M1. Low values in general point out transition zones between gaps and vegetation.*

*Even though V_M2 and Sten_M/O are tested to have evolved different vegetation structure, these maps show that the parts of V_M2 that had a fast colonization of Acer plantanous are overall similar to Sten_M/O.*
