# Supplementary material for: Using lidar to assess the development of structural diversity in forests undergoing passive rewilding in temperate Northern Europe
Source: PeerJ. 2019 Jan 14;6:e6219. doi: 10.7717/peerj.6219 (PMC6336013; doi:10.7717/peerj.6219)
Supplement: Supplemental Information 3 [file peerj-07-6219-s003.pdf]

PeerJ

Using lidar to assess the development of structural diversity in forests undergoing passive rewilding in temperate Northern Europe

Henrik Thers Peder Klith Bøcher, Jens-Christian Svenning

Table S1. Details on the 25 lidar-derived metrics used in this paper.

Metric	Explanation	Example of literature where the metric is used
CanopyCover	percentage of vegetation returns; the number of vegetation returns (no ground returns) divided by the number of all returns x 100	(Hudak et al. 2008)
CanopyHeight	the altitude value for the upper most return	(Drake et al. 2002; Goetz et al. 2007)
Percentile50_FirstReturn	the height from ground level that contains the lowest 50% of the first returns	(Næsset and Gobakken 2005)
Percentile75_FirstReturn	the height from ground level that contains the lowest 75% of the first returns	(Nord-Larsen and Schumacher, 2012)
Percentile95_FirstReturn	the height from ground level that contains the lowest 95% of the first returns	(Nord-Larsen and Schumacher, 2012)
GROUND	the intensity from the lowest 30 cm divided by the rest of the intensity	(Drake et al., 2002)
Height_(Z-Range)	the range (meters) between the lower most and upper most return	(Hudak et al., 2008)
Percentile50_All	the height from ground level that contains the lowest 50% of all returns	(Hudak et al. 2008; Sullivan et al. 2014)
Percentile75_All	the height from ground level that contains the lowest 75% of all returns	(Hudak et al., 2008; Sullivan et al., 2014)
Percentile90_All	the height from ground level that contains the lowest 90% of all returns	(Hudak et al., 2008; Sullivan et al., 2014)
Percentile95_All	the height from ground level that contains the lowest 95% of all returns	(Hudak et al., 2008; Sullivan et al., 2014)
Int.MEAN_above1m	mean intensity for returns with an altitude ≥ 1 meter	(Alexander et al. 2014)
Intensity_Skewness	a skewness calculation based on the intensity of all points	(Hudak et al., 2008)
MeanHeight_FirstReturn_above1m	the mean height for first returns above one meter	(Nord-Larsen and Schumacher, 2012)
Percent_SecondReturns	the percentage of second returns of all returns (first and second)	(Hudak et al., 2008)
Percentile05_ExclGround	the height from ground level that contains the lowest 5% of the vegetation returns (no ground returns)	
Percentile10_ExclGround	the height from ground level that contains the lowest 10% of the vegetation returns (no ground returns)	
Percentile25_ExclGround	the height from ground level that contains the lowest 25% of the vegetation returns (no ground returns)	
Percentile50_ExclGround	the height from ground level that contains the lowest 50% of the vegetation returns (no ground returns)	
Percentile75_ExclGround	the height from ground level that contains the lowest 75% of the vegetation returns (no ground returns)	

Percentile90_ExclGround	the height from ground level that contains the lowest 90% of the vegetation returns (no ground returns)	
Percentile95_ExclGround	the height from ground level that contains the lowest 95% of the vegetation returns (no ground returns)	
RelativeMeanHeight_All	the mean height for all returns divided by the height of the upper most return	(Nord-Larsen and Schumacher, 2012)
RelativeMeanHeight_above1m	the mean height for first returns above one meter divided by the upper most first return	(Nord-Larsen and Schumacher, 2012)
STRATUM1	percentage of non-ground returns; returns > 0 meter and ≤ 1 meter height, divided by all vegetation returns (no ground returns) x 100	(Hudak et al., 2008)

The 25 lidar metrics used in this paper listed with explanation for calculations along with examples of papers where the metrics have been used. If no reference is listed the authors have not seen the metric being used in existing papers.

Alexander C, Bøcher PK, Arge L, Svenning J-C (2014) Regional-scale mapping of tree cover, height and main phenological tree types using airborne laser scanning data. Remote Sens Environ 147:156–172 . doi: 10.1016/j.rse.2014.02.013

Drake JB, Dubayah RO, Clark DB, et al (2002) Estimation of tropical forest structural characteristics using large-footprint lidar. Remote Sens Environ 79:305–319 . doi: 10.1016/S0034-4257(01)00281-4

Goetz S, Steinberg D, Dubayah R, Blair B (2007) Laser remote sensing of canopy habitat heterogeneity as a predictor of bird species richness in an eastern temperate forest, USA. Remote Sens Environ 108:254–263 . doi: 10.1016/j.rse.2006.11.016

Hudak AT, Crookston NL, Evans JS, et al (2008) Nearest neighbor imputation of species-level, plot-scale forest structure attributes from LiDAR data. Remote Sens Environ 112:2232–2245 . doi: 10.1016/j.rse.2007.10.009

Næsset E, Gobakken T (2005) Estimating forest growth using canopy metrics derived from airborne laser scanner data. Remote Sens Environ 96:453–465 . doi: 10.1016/J.RSE.2005.04.001

Sullivan FB, Palace M, Ducey M (2014) Multivariate statistical analysis of asynchronous lidar data and vegetation models in a neotropical forest. Remote Sens Environ 154:368–377 . doi: 10.1016/j.rse.2014.04.027
