# Supplementary material for: Using lidar to assess the development of structural diversity in forests undergoing passive rewilding in temperate Northern Europe
Source: PeerJ. 2019 Jan 14;6:e6219. doi: 10.7717/peerj.6219 (PMC6336013; doi:10.7717/peerj.6219)
Supplement: Supplemental Information 4 [file peerj-07-6219-s004.pdf]

PeerJ

Using lidar to assess the development of structural diversity in forests undergoing passive rewilding in temperate Northern Europe

Henrik Thers, Peder Klith Bøcher, Jens-Christian Svenning

Table S2. Results from pairwise tests of medians and variance of PCA_10m values between all forests and zones.

area	axis	variance (σ^2)	V_O1	V_O2	V_O3	Sten_M/O	V_M2	V_M1	V_Y1	V_Y2	Tran_Y3	Fløj_Y4
**V_O1**	**Dissim.**			7	4	8	6	7	7	6	9	6
**V_O1**	**PC1**	6.58	x	*** / +++	+	*** / +++	***	***	***	***	*** / +++	*** / +++
**V_O1**	**PC2**	0.57	x	***	non-sig.	non-sig.	*** / +	*** / +++	*** / +++	*** / +++	*** / +++	***
**V_O1**	**PC3**	1.66	x	*** / +++	***	*** / +++	***	*** / +++	*** / +++	*** / +++	*** / +++	***
**V_O1**	**PC4**	0.76	x	+++	*	* / +++	*** / +++	***	+++	non-sig.	+++	*** / +++
**V_O1**	**PC5**	0.24	x	***	***	*** / +	non-sig.	+++	+++	+++	*** / +++	non-sig.
**V_O2**	**Dissim.**		7		7	7	9	8	9	6	9	7
**V_O2**	**PC1**	2.07	*** / +++	x	***	*** / +	*** / +++	*** / +++	*** / +++	*** / +++	*** / +++	*** / +
**V_O2**	**PC2**	0.66	***	x	***	***	***	* / +++	*** / +++	+++	*** / +++	*** / +++
**V_O2**	**PC3**	0.47	*** / +++	x	*** / +++	*** / +++	*** / +++	non-sig.	*** / +++	***	*** / +++	***
**V_O2**	**PC4**	0.10	+++	x	*** / +	+++	*** / +++	*** / +++	+++	+++	*** / +++	***
**V_O2**	**PC5**	0.21	***	x	***	***	*** / +	* / +++	*** / +++	+++	+++	**
**V_O3**	**Dissim.**		4	7		2	2	6	5	5	8	3
**V_O3**	**PC1**	1.60	+	***	x	non-sig.	*** / +	***	*** / ++	*** / ++	*** / +++	***
**V_O3**	**PC2**	0.24	non-sig.	***	x	non-sig.	non-sig.	***	++	***	***	***
**V_O3**	**PC3**	2.81	***	*** / +++	x	non-sig.	non-sig.	*** / +++	++	+++	+++	non-sig.
**V_O3**	**PC4**	0.35	*	*** / +	x	***	non-sig.	***	non-sig.	non-sig.	*** / +++	non-sig.
**V_O3**	**PC5**	0.16	***	***	x	***	***	***	+++	***	*** / +++	***
**Sten_M/O**	**Dissim.**		8	7	2		9	10	9	10	9	8
**Sten_M/O**	**PC1**	3.59	*** / +++	*** / +	non-sig.	x	*** / +++	*** / +++	*** / +++	*** / +++	*** / +++	*** / +++
**Sten_M/O**	**PC2**	1.06	non-sig.	***	non-sig.	x	***	*** / +++	*** / +++	*** / +++	*** / +++	***
**Sten_M/O**	**PC3**	2.42	*** / +++	*** / +++	non-sig.	x	*** / +++	*** / +++	*** / +++	*** / +++	*** / +++	*** / +++
**Sten_M/O**	**PC4**	1.99	* / +++	+++	***	x	*** / +++	*** / +++	***	* / ++	***	*** / +++
**Sten_M/O**	**PC5**	0.16	*** / +	***	***	x	*** / +++	*** / +++	*** / +++	*** / +++	*** / +++	***
**V_M2**	**Dissim.**		6	9	3	9		9	7	9	10	7
**V_M2**	**PC1**	7.48	***	*** / +++	*** / +	*** / +++	x	*** / +	***	***	*** / +++	*** / +++
**V_M2**	**PC2**	0.85	*** / +	***	non-sig.	***	x	*** / +++	+++	*** / +++	*** / +++	* / +++
**V_M2**	**PC3**	1.46	***	*** / +++	non-sig.	*** / +++	x	*** / +++	*** / +++	*** / +++	*** / +++	***
**V_M2**	**PC4**	0.55	*** / +++	*** / +++	non-sig.	*** / +++	x	*** / +++	** / +	*** / +++	*** / +++	* / ++
**V_M2**	**PC5**	0.30	non-sig.	*** / *	***	*** / +++	x	+++	+++	* / +++	*** / +++	non-sig.
**V_M1**	**Dissim.**		7	8	6	10	9		8	4	9	8
**V_M1**	**PC1**	6.32	***	*** / +++	***	*** / +++	*** / +	x	***	***	*** / +++	*** / +++
**V_M1**	**PC2**	3.12	*** / +++	* / +++	***	*** / +++	*** / +++	x	*** / +++	***	+++	*** / +++
**V_M1**	**PC3**	0.66	*** / +++	non-sig.	*** / +++	*** / +++	*** / +++	x	*** / +++	***	*** / +++	***
**V_M1**	**PC4**	0.79	***	*** / +++	***	*** / +++	*** / +++	x	*** / +++	***	*** / +++	*** / +++
**V_M1**	**PC5**	1.28	+++	* / +++	***	*** / +++	+++	x	+++	non-sig.	*** / +++	+
**V_Y1**	**Dissim.**		7	9	5	9	7	8		6	10	7
**V_Y1**	**PC1**	5.91	***	*** / +++	*** / ++	*** / +++	***	***	x	***	*** / +++	*** / +++
**V_Y1**	**PC2**	4.32	*** / +++	*** / +++	++	*** / +++	+++	*** / +++	x	*** / +	*** / +++	+++
**V_Y1**	**PC3**	0.90	*** / +++	*** / +++	++	*** / +++	*** / +++	*** / +++	x	non-sig.	*** / +++	***
**V_Y1**	**PC4**	1.54	+++	+++	non-sig.	***	** / +	*** / +++	x	++	* / +++	*** / +++
**V_Y1**	**PC5**	2.33	+++	*** / +++	+++	*** / +++	+++	+++	x	* / +++	*** / +++	+++

Table S2 continued

area	axis	variance (σ²)	V_O1	V_O2	V_O3	Sten_M/O	V_M2	V_M1	V_Y1	V_Y2	Tran_Y3	Fløj_Y4
**V_Y2**	**Dissim.**		6	6	5	10	9	4	6		9	7
**V_Y2**	**PC1**	6.08	***	*** / +++	*** / ++	*** / +++	***	***	***	x	*** / +++	*** / +++
**V_Y2**	**PC2**	3.31	*** / +++	+++	***	*** / +++	*** / +++	***	*** / +	x	*** / +++	+++
**V_Y2**	**PC3**	0.69	*** / +++	***	+++	*** / +++	*** / +++	***	non-sig.	x	*** / +++	***
**V_Y2**	**PC4**	0.95	non-sig.	+++	non-sig.	* / ++	*** / +++	***	++	x	+++	*** / +++
**V_Y2**	**PC5**	1.30	+++	+++	***	*** / +++	* / +++	non-sig.	* / +++	x	* / +++	+++
**Tran_Y3**	**Dissim.**		9	9	8	9	10	9	10	9		8
**Tran_Y3**	**PC1**	0.27	*** / +++	*** / +++	*** / +++	*** / +++	*** / +++	*** / +++	*** / +++	*** / +++	x	*** / +++
**Tran_Y3**	**PC2**	0.16	*** / +++	*** / +++	***	*** / +++	*** / +++	+++	*** / +++	*** / +++	x	***
**Tran_Y3**	**PC3**	0.24	*** / +++	*** / +++	+++	*** / +++	*** / +++	*** / +++	*** / +++	*** / +++	x	+++
**Tran_Y3**	**PC4**	0.05	+++	*** / +++	*** / +++	+++	*** / +++	*** / +++	* / +++	+++	x	*** / +++
**Tran_Y3**	**PC5**	0.04	*** / +++	+++	*** / +++	*** / +++	*** / +++	*** / +++	*** / +++	* / +++	x	*** / +++
**Fløj_Y4**	**Dissim.**		6	7	3	8	7	8	7	7	8	
**Fløj_Y4**	**PC1**	0.66	*** / +++	*** / +	***	*** / +++	*** / +++	*** / +++	*** / +++	*** / +++	*** / +++	x
**Fløj_Y4**	**PC2**	0.22	***	*** / +++	***	***	* / +++	*** / +++	+++	+++	***	x
**Fløj_Y4**	**PC3**	0.92	***	***	non-sig.	*** / +++	***	***	***	***	+++	x
**Fløj_Y4**	**PC4**	0.17	*** / +++	***	non-sig.	*** / +++	* / ++	*** / +++	*** / +++	*** / +++	*** / +++	x
**Fløj_Y4**	**PC5**	0.16	non-sig.	**	***	***	non-sig.	+	+++	+++	*** / +++	x

*All managed forests and zones of Vorsø were tested pairwise according to medians and variance of the first five axes of the PCA_10m (PCA of all 10-meter cells in the seven Vorsø zones and the three managed forests). We used Kruskal-Wallis to test for different medians and Levene's test to test for different variances. Asterisks indicate significance for medians and plusses indicate significance for variance. Non-sig. indicates non-significance of both medians and variance. Significance levels have been Bonferroni corrected by dividing 0.05 with 225. The corrected level of significance used here was 0.000222. */+ = below 0.000222, **/++ = below 0.00001, ***/+++ = below 0.000001. The column of variance (σ²) lists variance values.* The rows marked *dissim.* list how many of the 10 pairwise tests are significant as a measure of *dissimilarity* between two areas.
