# Supplementary material for: Using lidar to assess the development of structural diversity in forests undergoing passive rewilding in temperate Northern Europe
Source: PeerJ. 2019 Jan 14;6:e6219. doi: 10.7717/peerj.6219 (PMC6336013; doi:10.7717/peerj.6219)
Supplement: Supplemental Information 5 [file peerj-07-6219-s005.pdf]

PeerJ

Using lidar to assess the development of structural diversity in forests undergoing passive rewilding in temperate Northern Europe

Henrik Thers, Peder Klith Bøcher, Jens-Christian Svenning

Table S3. Results from pairwise tests of medians and variance of PCA_30m values between all forests and zones.

area	axis	variance (σ^2)	V_O1	V_O2	V_O3	Sten_M/O	V_M2	V_M1	V_Y1	V_Y2	Tran_Y3	Fløj_Y4
**V_O1**	**Dissim.**			4	0	7	2	9	6	7	10	3
**V_O1**	**PC1**	21.63	x	***	non-sign.	*** / +++	***	*** / +++	***	***	*** / +++	***
**V_O1**	**PC2**	1.55	x	***	non-sign.	*	non-sign.	*** / +	***	***	*** / +++	*
**V_O1**	**PC3**	1.85	x	non-sign.	non-sign.	***	non-sign.	***	***	***	*** / +++	non-sign.
**V_O1**	**PC4**	0.41	x	***	non-sign.	***	non-sign.	*** / ++	+++	*** / +	*** / +++	*
**V_O1**	**PC5**	2.78	x	***	non-sign.	*** / +++	++	* / +++	*** / +++	*** / +++	** / +++	non-sign.
**V_O2**	**Dissim.**		4		0	2	4	5	5	4	5	3
**V_O2**	**PC1**	8.94	***	x	non-sign.	non-sign.	non-sign.	non-sign.	non-sign.	non-sign.	*** / +++	**
**V_O2**	**PC2**	0.82	***	x	non-sign.	***	***	** / +	*** / +	* / +	+++	**
**V_O2**	**PC3**	0.58	non-sign.	x	non-sign.	***	***	*** / +	***	***	*	non-sign.
**V_O2**	**PC4**	0.28	***	x	non-sign.	non-sign.	*	non-sign.	+++	non-sign.	non-sign.	non-sign.
**V_O2**	**PC5**	0.55	***	x	non-sign.	non-sign.	***	***	*	***	***	***
**V_O3**	**Dissim.**		0	0		0	0	0	0	0	0	0
**V_O3**	**PC1**	0.56	non-sign.	non-sign.	x	non-sign.	non-sign.	non-sign.	non-sign.	non-sign.	non-sign.	non-sign.
**V_O3**	**PC2**	0.39	non-sign.	non-sign.	x	non-sign.	non-sign.	non-sign.	non-sign.	non-sign.	non-sign.	non-sign.
**V_O3**	**PC3**	0.37	non-sign.	non-sign.	x	non-sign.	non-sign.	non-sign.	non-sign.	non-sign.	non-sign.	non-sign.
**V_O3**	**PC4**	0.32	non-sign.	non-sign.	x	non-sign.	non-sign.	non-sign.	non-sign.	non-sign.	non-sign.	non-sign.
**V_O3**	**PC5**	0.42	non-sign.	non-sign.	x	non-sign.	non-sign.	non-sign.	non-sign.	non-sign.	non-sign.	non-sign.
**Sten_M/O**	**Dissim.**		7	2	0		7	10	8	8	10	4
**Sten_M/O**	**PC1**	3.52	*** / +++	non-sign.	non-sign.	x	***	*** / +	+	** / +	*** / ++	*
**Sten_M/O**	**PC2**	1.03	*	***	non-sign.	x	*** / +++	*** / +++	*** / +++	*** / +++	*** / +++	***
**Sten_M/O**	**PC3**	1.39	***	***	non-sign.	x	***	*** / +++	***	***	*** / +++	***
**Sten_M/O**	**PC4**	0.44	***	non-sign.	non-sign.	x	*** / +	*** / +++	** / +++	+++	** / ++	non-sign.
**Sten_M/O**	**PC5**	0.80	*** / +++	non-sign.	non-sign.	x	***	*** / +	*** / ++	*** / ++	*** / +++	***
**V_M2**	**Dissim.**		2	4	0	7		4	4	4	9	1
**V_M2**	**PC1**	8.65	***	non-sign.	non-sign.	***	x	non-sign.	non-sign.	non-sign.	*** / +++	***
**V_M2**	**PC2**	3.93	non-sign.	***	non-sign.	*** / +++	x	***	***	***	*** / +++	non-sign.
**V_M2**	**PC3**	1.07	non-sign.	***	non-sign.	***	x	*** / ++	***	*	*** / +++	non-sign.
**V_M2**	**PC4**	0.98	non-sign.	*	non-sign.	*** / +	x	***	++	*	*** / +++	non-sign.
**V_M2**	**PC5**	0.91	++	***	non-sign.	***	x	non-sign.	***	*	+	non-sign.
**V_M1**	**Dissim.**		9	5	0	10	4		4	0	8	5
**V_M1**	**PC1**	6.09	*** / +++	non-sign.	non-sign.	*** / +	non-sign.	x	*	non-sign.	*** / +++	*** / +
**V_M1**	**PC2**	3.57	*** / +	** / +	non-sign.	*** / +++	***	x	non-sign.	non-sign.	*** / +++	*** / ++
**V_M1**	**PC3**	2.58	***	*** / +	non-sign.	*** / +++	*** / ++	x	non-sign.	non-sign.	*** / +++	***
**V_M1**	**PC4**	1.68	*** / ++	non-sign.	non-sign.	*** / +++	***	x	*** / +	non-sign.	+++	non-sign.
**V_M1**	**PC5**	1.68	* / +++	***	non-sign.	*** / +	non-sign.	x	**	non-sign.	+	non-sign.
**V_Y1**	**Dissim.**		6	5	0	8	4	4		0	8	6
**V_Y1**	**PC1**	7.55	***	non-sign.	non-sign.	+	non-sign.	*	x	non-sign.	*** / +++	*
**V_Y1**	**PC2**	3.15	***	*** / +	non-sign.	*** / +++	***	non-sign.	x	non-sign.	*** / +++	*** / +
**V_Y1**	**PC3**	1.56	***	***	non-sign.	***	***	non-sign.	x	non-sign.	*** / +++	***
**V_Y1**	**PC4**	2.38	+++	+++	non-sign.	** / +++	++	*** / +	x	non-sign.	** / +++	+++
**V_Y1**	**PC5**	0.28	*** / +++	*	non-sign.	*** / ++	***	**	x	non-sign.	non-sign.	*

Table S3 continued

area	axis	variance (σ²)	V_O1	V_O2	V_O3	Sten_M/O	V_M2	V_M1	V_Y1	V_Y2	Tran_Y3	Fløj_Y4
**V_Y2**	**Dissim.**		7	4	0	8	4	0	0		7	5
**V_Y2**	**PC1**	6.88	***	non-sign.	non-sign.	** / +	non-sign.	non-sign.	non-sign.	x	*** / +++	*** / +
**V_Y2**	**PC2**	2.88	***	* / +	non-sign.	*** / +++	***	non-sign.	non-sign.	x	*** / +++	*** / ++
**V_Y2**	**PC3**	1.56	***	***	non-sign.	***	*	non-sign.	non-sign.	x	*** / +++	**
**V_Y2**	**PC4**	2.49	*** / +	non-sign.	non-sign.	+++	*	non-sign.	non-sign.	x	+++	non-sign.
**V_Y2**	**PC5**	0.20	*** / +++	***	non-sign.	*** / ++	*	non-sign.	non-sign.	x	non-sign.	non-sign.
**Tran_Y3**	**Dissim.**		10	5	0	10	9	8	8	7		3
**Tran_Y3**	**PC1**	1.00	*** / +++	*** / +++	non-sign.	*** / ++	*** / +++	*** / +++	*** / +++	*** / +++	x	***
**Tran_Y3**	**PC2**	0.07	*** / +++	+++	non-sign.	*** / +++	*** / +++	*** / +++	*** / +++	*** / +++	x	***
**Tran_Y3**	**PC3**	0.29	*** / +++	*	non-sign.	*** / +++	*** / +++	*** / +++	*** / +++	*** / +++	x	*
**Tran_Y3**	**PC4**	0.09	*** / +++	non-sign.	non-sign.	** / ++	*** / +++	+++	** / +++	+++	x	non-sign.
**Tran_Y3**	**PC5**	0.19	** / +++	***	non-sign.	*** / +++	+	+	non-sign.	non-sign.	x	non-sign.
**Fløj_Y4**	**Dissim.**		3	3	0	4	1	5	6	5	3	
**Fløj_Y4**	**PC1**	0.65	***	**	non-sign.	*	***	*** / +	*	*** / +	***	x
**Fløj_Y4**	**PC2**	0.23	*	**	non-sign.	***	non-sign.	*** / ++	*** / +	*** / ++	***	x
**Fløj_Y4**	**PC3**	0.74	non-sign.	non-sign.	non-sign.	***	non-sign.	***	***	**	*	x
**Fløj_Y4**	**PC4**	0.16	*	non-sign.	non-sign.	non-sign.	non-sign.	non-sign.	+++	non-sign.	non-sign.	x
**Fløj_Y4**	**PC5**	0.35	non-sign.	***	non-sign.	***	non-sign.	non-sign.	*	non-sign.	non-sign.	x

*All managed forests and zones of Vorsø were tested pairwise according to medians and variance of the first five axes of the PCA_30m (PCA of all 30-meter cells in the seven Vorsø zones and the three managed forests). We used Kruskal-Wallis to test for different medians and Levene's test to test for different variances. Asterisks indicate significance for medians and plusses indicate significance for variance. Non-sig. indicates non-significance of both medians and variance. Significance levels have been Bonferroni corrected by dividing 0.05 with 225. The corrected level of significance used here was 0.000222. */+ = below 0.000222, **/++ = below 0.00001, ***/+++ = below 0.000001. The column of variance (σ²) lists variance values.* The rows marked *dissim.* list how many of the 10 pairwise tests are significant as a measure of *dissimilarity* between two areas.
