# Supplementary material for: Using lidar to assess the development of structural diversity in forests undergoing passive rewilding in temperate Northern Europe
Source: PeerJ. 2019 Jan 14;6:e6219. doi: 10.7717/peerj.6219 (PMC6336013; doi:10.7717/peerj.6219)
Supplement: Supplemental Information 6 [file peerj-07-6219-s006.pdf]

PeerJ

Using lidar to assess the development of structural diversity in forests undergoing passive rewilding in temperate Northern Europe

Henrik Thers, Peder Klith Bøcher, Jens-Christian Svenning

Table S4. Interpretation of axes.

PCA_10m	Interpretation
Axis 1	Expresses a gradient of vegetation height (Fig. 3a). V_M2 shows the highest variance on this axis due to the combination of both tall-stemmed trees and low and open vegetation. The V_O1 has the second-highest variance due to the moderate disturbance conditions (Halberg and Gregersen, 2010), which have created a mosaic of tall mature trees and gaps.
Axis 2	Represents a gradient from open areas (low values) to lower dense vegetation (high values). Taller broadleaved vegetation has intermediate values. On Vorsø the highest scoring cells contain blackthorn scrub and in Sten_M/O the highest values belong to cells holding a stand of spruce (Fig. S2).
Axis 3	Expresses a gradient that assigns low values to cells with a large proportion of vegetative returns compared to ground returns, in combination with a dense understory – especially below one meter. The gradient assigns high values to taller vegetation with a large proportion of returns concentrated in the canopy layer. The V_O1 has a significantly lower median compared to all other forests and zones (Fig. 3), due to the open canopy layer entailing increased light to the forest floor.
Axis 4	The median height of all returns (Percentile50_All) dominate this axis together with the relative height metrics and GROUND. The very dense vegetation can ignore the negative loading of GROUND since dense vegetation has low values on this metric. The conifer stand of Sten_M/O score the highest values of this axis while low vegetation and tall stemmed broadleaved vegetation in general score low values.
Axis 5	The STRATUM1 metric dominates this axis, entailing a gradient from open areas to low sparse vegetation. All other vegetation types are assigned intermediate values. STRATUM1 subscribes the highest values to cells with all returns between 0 and 1 meter of height, and the lowest values to cells without vegetation.
PCA_30m	
Axis 1	Represents a simple gradient of heterogeneity of height subscribing low values to heterogeneous cells (30 × 30-meter) and high values to homogeneous cells. The V_O1 shows a median value significantly lower than all other areas except the V_O3 (assumable due to lack of statistical power because of too few cells (Fig. 3 and Table S2 and S3)).
Axis 2	This axis is difficult to interpret. Overall, it assigns high values to cells containing edges between low and no vegetation and are reserved for the V_M1, V_Y1 and V_Y2, and low values to heterogeneous areas containing taller vegetation. According to this, cells with values around zero should be homogenous, or it could be due to a special combination of negative and positive loadings.
Axis 3	Subscribes the lowest values to heterogeneous vegetation according to openness. This gradient points out some locations in the V_M1 (by extreme low values), which have evolved a unique structure that the younger V_Y1 and V_Y2 cannot resemble (Fig. 3h Fig. S2), as well as some cells in Sten_M/O on the edge between spruce and beech.

Axis 4	This is another axis where both high and low values present heterogeneous vegetation. Heterogeneity related to vegetation density is assigned high values and heterogeneity related to sparse or no vegetation is assigned low values, which entails the largest variance values belongs to the V_M1, V_Y1 and V_Y2.
Axis 5	This axis also belongs to the complex axes. Heterogeneity related to vegetation height is assigned low values and heterogeneity related to density of returns in the lower part of the vegetation is assigned high values, which entails that the V_O1 shows a large variance since it contains both these structural attributes.
