# Supplementary material for: Using lidar to assess the development of structural diversity in forests undergoing passive rewilding in temperate Northern Europe
Source: PeerJ. 2019 Jan 14;6:e6219. doi: 10.7717/peerj.6219 (PMC6336013; doi:10.7717/peerj.6219)
Supplement: Supplemental Information 7 [file peerj-07-6219-s007.pdf]

PeerJ

Using lidar to assess the development of structural diversity in forests undergoing passive rewilding in temperate Northern Europe

Henrik Thers, Peder Klith Bøcher, Jens-Christian Svenning

Table S5. Eigenvectors of the first five axes of PCA_10m.

Metrics\Axes	PC1	PC2	PC3	PC4	PC5
Proportion of Variance	0.654	0.088	0.073	0.048	0.032
Cumulative Proportion	0.654	0.742	0.813	0.860	0.892
CanopyCover	0.149	**0.368**	**-0.300**	0.193	**-0.212**
CanopyHeight	**0.237**	-0.077	-0.111	-0.063	-0.014
Percentile50_FirstReturn	**0.222**	-0.036	-0.006	0.164	0.045
Percentile75_FirstReturn	**0.240**	-0.087	-0.065	0.010	0.011
Percentile95_FirstReturn	**0.239**	-0.086	-0.103	-0.047	-0.015
GROUND	-0.118	**-0.460**	0.094	**0.390**	-0.114
Height_(Z-Range)	**0.237**	-0.077	-0.111	-0.064	-0.014
Percentile50_All	0.111	0.170	-0.024	***0.666***	0.034
Percentile75_All	**0.235**	-0.037	-0.009	0.121	0.045
Percentile90_All	**0.241**	-0.083	-0.079	-0.017	0.011
Percentile95_All	**0.240**	-0.084	-0.094	-0.041	0.000
Int.MEAN_above1m	-0.071	**0.498**	-0.139	0.106	**0.251**
Intensity_Skewness	0.157	-0.076	-0.071	0.176	-0.023
MeanHeight_FirstReturn_above1m	**0.242**	-0.081	0.000	-0.047	0.099
Percent_SecondReturns	**0.211**	0.063	-0.108	-0.163	**-0.253**
Percentile05_ExclGround	0.120	0.091	***0.579***	0.096	0.078
Percentile10_ExclGround	0.162	0.060	***0.512***	0.111	0.096
Percentile25_ExclGround	**0.213**	-0.015	**0.280**	0.034	0.126
Percentile50_ExclGround	**0.235**	-0.072	0.085	-0.035	0.112
Percentile75_ExclGround	**0.241**	-0.093	-0.034	-0.058	0.057
Percentile90_ExclGround	**0.240**	-0.092	-0.081	-0.066	0.021
Percentile95_ExclGround	**0.239**	-0.087	-0.095	-0.065	0.007
RelativeMeanHeight_All	0.179	**0.356**	-0.040	**0.210**	-0.109
RelativeMeanHeight_above1m	0.134	**0.339**	0.148	**-0.375**	**0.319**
STRATUM1	-0.090	-0.183	**-0.294**	0.146	***0.799***

The explained variance and eigenvectors for the first five axes of PCA_10m based on 25 lidar metrics computed in 10-meter cells in three managed forests and seven zones of Vorsø (a total of 7319 cells). Loadings above 0.2 are highlighted in red and loadings above 0.5 are highlighted in orange and italics.
