# Supplementary material for: Using lidar to assess the development of structural diversity in forests undergoing passive rewilding in temperate Northern Europe
Source: PeerJ. 2019 Jan 14;6:e6219. doi: 10.7717/peerj.6219 (PMC6336013; doi:10.7717/peerj.6219)
Supplement: Supplemental Information 8 [file peerj-07-6219-s008.pdf]

PeerJ

Using lidar to assess the development of structural diversity in forests undergoing passive rewilding in temperate Northern Europe

Henrik Thers, Peder Klith Bøcher, Jens-Christian Svenning

Table S6. Eigenvectors of the first five axes of PCA_30m.

Metrics\Akse	PC1	PC2	PC3	PC4	PC5
Proportion of Variance	0.428	0.254	0.109	0.053	0.036
Cumulative Proportion	0.428	0.682	0.791	0.843	0.880
CanopyCover	-0.115	0.198	**-0.341**	**0.373**	**-0.203**
CanopyHeight	**-0.271**	0.117	0.083	0.046	**0.249**
Percentile50_FirstReturn	-0.150	-0.290	-0.089	-0.047	**-0.321**
Percentile75_FirstReturn	**-0.270**	-0.102	0.063	0.051	-0.185
Percentile95_FirstReturn	**-0.287**	0.076	0.087	0.071	0.182
GROUND	-0.007	**0.296**	**-0.233**	**-0.365**	0.009
Percentile50_All	-0.054	**-0.256**	**-0.210**	0.092	**0.222**
Percentile75_All	**-0.201**	**-0.243**	-0.075	-0.034	**-0.337**
Percentile90_All	**-0.297**	-0.027	0.056	0.004	-0.015
Percentile95_All	**-0.295**	0.045	0.076	0.021	0.113
Height_(Z-Range)	**-0.271**	0.117	0.081	0.051	**0.252**
Int.MEAN_above1m	-0.032	**0.294**	**-0.242**	-0.133	-0.022
Intensity_Skewness	0.016	-0.102	**-0.387**	0.093	**0.408**
MeanHeight_FirstReturn_above1m	**-0.278**	-0.084	-0.013	**-0.211**	-0.057
Percent_SecondReturns	-0.176	0.150	**-0.206**	**0.360**	-0.036
Percentile05_ExclGround	0.021	**-0.298**	**-0.264**	-0.062	**0.289**
Percentile10_ExclGround	-0.008	**-0.308**	**-0.296**	-0.099	**0.203**
Percentile25_ExclGround	-0.075	**-0.289**	**-0.256**	-0.189	-0.031
Percentile50_ExclGround	**-0.233**	-0.179	-0.029	**-0.235**	**-0.241**
Percentile75_ExclGround	**-0.287**	-0.051	0.090	-0.132	-0.046
Percentile90_ExclGround	**-0.291**	0.035	0.117	-0.031	0.153
Percentile95_ExclGround	**-0.285**	0.063	0.115	0.010	**0.209**
RelativeMeanHeight_All	-0.139	0.135	**-0.356**	**0.370**	**-0.244**
RelativeMeanHeight_above1m	-0.064	**0.306**	**-0.206**	**-0.372**	-0.055
STRATUM1	-0.042	**0.270**	**-0.244**	**-0.325**	-0.013

The explained variance and eigenvectors for the first five axes of PCA_30m based on 25 lidar metrics computed in 30-meter cells in three managed forests and seven zones of Vorsø (a total of 1002 cells). Loadings above 0.2 are highlighted in red. It is important to note that the PCA_30m is based on the SD value between each cell (10 × 10-meters) and the eight surrounding cells, which has afterwards been collapsed into 30 × 30-meters cells. This means that the input is not the values of the original metrics listed in Table S1.
