# Supplementary material for: Using lidar to assess the development of structural diversity in forests undergoing passive rewilding in temperate Northern Europe
Source: PeerJ. 2019 Jan 14;6:e6219. doi: 10.7717/peerj.6219 (PMC6336013; doi:10.7717/peerj.6219)
Supplement: Supplemental Information 9 [file peerj-07-6219-s009.docx]

PeerJ

Using lidar to assess the development of structural diversity in forests undergoing passive rewilding in temperate Northern Europe

Henrik Thers, Peder Klith Bøcher, Jens-Christian Svenning

Raw data:

Lidar data used in this study is included in the below files freely available at the <https://download.kortforsyningen.dk/content/dhm-2007punktsky> homepage.

punktsky_619_55_LAS_UTM32-EUREF89.ZIP

punktsky_621_57_LAS_UTM32-EUREF89.ZIP

punktsky_621_56_LAS_UTM32-EUREF89.ZIP

punktsky_619_56_LAS_UTM32-EUREF89.ZIP

punktsky_622_57_LAS_UTM32-EUREF89.ZIP
